# Limitations in US Cancer Screening Rate Data and Opportunities to Improve Precision Prevention

**DOI:** 10.3390/diagnostics16193089

**Published:** 2026-09-24

**Authors:** Jody Hoyos, Elissa Quinn, Daryl Pritchard, Gary Gustavsen, Owen Fahey, Arushi Agarwal, Chyke Doubeni

**Affiliations:** 1 Prevent Cancer Foundation, 333 John Carlyle Street, Suite 635, Alexandria, VA 22314, USA; jody.hoyos@preventcancer.org; 2 AstraZeneca, Wilmington, DE 19803, USA; elissa.quinn@astrazeneca.com; 3 Personalized Medicine Coalition, 1710 Rhode Island Ave NW, Washington, DC 20036, USA; dpritchard@personalizedmedicinecoalition.org; 4 Health Advances LLC, 275 Grove Street, Newton, MA 02466, USA; ofahey@healthadvances.com (O.F.); aagarwal@healthadvances.com (A.A.); 5 The Ohio State University Wexner Medical Center, 410 West Tenth Avenue, Columbus, OH 43210, USA; chyke.doubeni@osumc.edu

**Keywords:** cancer screening, screening data, early detection, real-world data, electronic health records, claims data, health disparities, data quality

## Abstract

**Background**: Cancer screening is central to cancer control and an increasingly important component of personalized medicine, enabling risk-adaptive prevention, earlier detection, and more tailored intervention strategies. However, screening participation varies widely across tumor types, populations, and geographic regions in the United States. Although screening rate data are widely used to guide clinical recommendations, policy, advocacy, and early detection innovation, the underlying data ecosystem remains fragmented and poorly characterized. Screening rates are commonly derived from surveys, electronic health records (EHRs), and claims data, each with limitations that may misrepresent true screening participation and hinder efforts to improve early detection. This study evaluated the current cancer screening data ecosystem to characterize key limitations and identify actionable solutions. **Methods:** Primary qualitative interviews were conducted with cancer screening data users (n = 8), including academic researchers and patient advocacy organizations, and data aggregators (n = 2) from national public health organizations. Secondary research included a structured review of publicly available survey-, EHR-, and claims-based screening data sources and relevant literature. Findings were synthesized and evaluated in a multistakeholder workshop with 20 participants representing data aggregators, researchers, clinicians, advocates, and industry experts to identify and prioritize solutions based on feasibility and potential impact. **Results:** Four major categories of limitations were identified across data sources: access, accuracy, consistency, and completeness. Data fragmentation, variable transparency, technical complexity, and cost limit accessibility and comparability. Accuracy is affected by recall bias, documentation and coding errors, and imperfect correction methods. Inconsistent tumor coverage, survey changes, and irregular reporting cycles hinder longitudinal analyses. Gaps in underserved population capture, risk factors, follow-up, and outcomes limit assessment of disparities and real-world impact. Stakeholders prioritized near-term solutions, including improved data education, standardized reporting, cross-source validation, and expanded data sharing, alongside longer-term investments in registries and longitudinal infrastructure. **Conclusions:** Structural limitations in cancer screening data constrain efforts to optimize screening, address inequities, and advance early detection. Targeted improvements and longer-term system investments are needed to strengthen data quality, usability, and population-level impact.

## 1. Introduction

Cancer screening is an important public health strategy aimed at detecting cancers at earlier stages to improve patient outcomes. It is central to cancer control and an increasingly important component of personalized medicine, enabling risk-adaptive prevention, earlier detection, and more tailored intervention strategies. Current guidelines recommend routine screening for several cancers, including breast, cervical, colorectal, and lung cancers based on evidence of improved patient outcomes (i.e., shifting diagnoses to earlier stages, thus detecting and treating earlier invasive lesions where curative therapy is more successful). However, screening participation varies widely across tumor types, populations, and geographies in the United States.

The current landscape of cancer screening data (i.e., the percentage of eligible people who actually get a recommended cancer screening test) is complex and has clear limitations. Despite ongoing efforts to enable and accelerate awareness and adoption of early cancer detection methods, numerous issues exist in the consistency and reporting of cancer screening rates [1,2,3].

Data on participation in cancer screening is primarily sourced from surveys, electronic health records (EHRs), or insurance claims and is used by various stakeholders to inform healthcare policy, clinical guidance, patient advocacy, or commercial research efforts to improve patient outcomes [4,5,6]. However, there are major gaps in the reported data across years, geographies, malignancies, and stages, with additional discrepancies arising due to differences in populations, sampling, and reporting methods [7,8]. Standards may not be in place to ensure quality and comparability across surveys with cancer screening components [9].

Cancer screening rate data is widely used by different stakeholders for diverse applications. Researchers, clinicians, policymakers, patient advocacy groups, and industry professionals all use cancer screening data for a range of purposes, all aimed at improving the utility, adherence, and impact of screening tools and protocols. The utility of accurate and detailed cancer screening rate data has far-reaching implications across personalized medicine. Applications include advocacy efforts around cancer screening policies, public education, and perhaps most central to personalized medicine, the prioritization and development of new cancer screening modalities. These applications place unique requirements on data quality (e.g., timeliness, granularity, representativeness), and any data limitations have practical consequences.

These limitations, which are often inherent to the underlying data source, significantly impair the ability to accurately measure screening participation, refine risk-adaptive screening recommendations, and assess relationships with downstream patient outcomes [10,11]. It is therefore critical to develop solutions to address these challenges and improve the utility of cancer screening data, including the establishment of more accurate baselines and more consistent reporting structures for up-to-date screening rates [12,13].

This study aims to evaluate the uses and limitations of cancer screening data derived from various sources. We also aim to identify opportunities to strengthen data systems to support efforts to inform patient care, public health decision-making, and innovation in averting preventable cancer deaths.

## 2. Materials and Methods

We employed a hybrid approach combining qualitative interviews with purposively selected opinion leaders in cancer screening and a targeted literature review informed by insights from interviews.

Interviews were conducted with eight data users and two data aggregators. We identified data users from academic researchers, Prevent Cancer Foundation, and patient advocacy groups such as the LUNGevity Early Lung Cancer Center. Data aggregators included experts from the American Cancer Society (ACS) and the Centers for Disease Control and Prevention (CDC). These interviewees were carefully selected based on their contributions to the field, publication history, and engagement in industry meetings. Interviews were guided by a topic guide focused on understanding how cancer screening data are accessed, interpreted, and applied across use cases, as well as perceived limitations in data availability, accuracy, consistency, and completeness. While these interviews were rich in content and broad in scope, we leveraged additional secondary research and a subsequent multistakeholder workshop to further expand on these learnings.

We conducted a structured review of publicly available cancer screening data resources, including sources from the ACS and CDC (which hosts the Behavioral Risk Factor Surveillance System) to document limitations related to data consistency and reporting clarity. A targeted review of published literature and conference proceedings was also conducted to identify evidence describing the strengths, limitations, and gaps in screening data sources. This search leveraged PubMed and other relevant databases and focused on research utilizing cancer screening rates derived from the major sources described in the literature. This review was not intended to be exhaustive but rather to provide a foundation for ongoing discussion.

The interviews and literature search results were synthesized to identify recurring themes and to categorize limitations and potential solutions across data source types. Finally, findings were evaluated in a multistakeholder workshop with 20 participants representing data aggregators, researchers, clinicians, advocates, and industry experts to identify and prioritize solutions based on feasibility and potential impact. Similar to the interviews, workshop participants were carefully selected based on their contributions to the field, publication history, and engagement in industry meetings. Feasibility and potential impact of solutions were assessed in real time and represented expert consensus as determined throughout the workshop.

## 3. Results

### 3.1. Overview of Cancer Screening Rate Data

From initial collection to public dissemination, cancer screening data pass through multiple stages, including data collection, processing, analysis, visualization, and reporting. At each stage, data may be modified, aggregated, or interpreted by different stakeholders, contributing to variability across published screening rate estimates.

Cancer screening data are generated and consolidated by a range of data aggregators, including government-funded research organizations, health systems, public payers, and commercial entities. Data sources include population-based surveys, electronic health records (EHRs), and insurance claims. Across all source types, data collection processes may be affected by incomplete capture, inconsistencies, and reporting errors. Subsequent processing steps may involve weighting to represent broader populations, correction for sampling bias, and imputation of missing values, with the goal of transforming raw data into analytic datasets or visualization tools such as dashboards or geographic maps.

Data users, including clinicians, health systems, academic researchers, patient advocacy organizations, policymakers, and industry stakeholders, analyze and publish findings derived from these datasets. Analyses often focus on specific tumor types, populations, or geographies and may involve linking multiple data sources, such as combining survey-based screening participation with registry-based incidence or mortality data. Outputs are disseminated through peer-reviewed publications, public reports, policy briefs, or commercial analyses.

The primary sources of cancer screening rate estimates are nationally sponsored surveys administered by organizations such as the Centers for Disease Control and Prevention (CDC) and the National Cancer Institute (NCI). Commonly used survey-based sources include the National Health Interview Survey Cancer Control Supplement (NHIS CSS), the Behavioral Risk Factor Surveillance System (BRFSS), and the Health Information National Trends Survey (HINTS). Complementary clinical data sources include aggregated EHR datasets, insurance claims, and structured reporting systems such as the Healthcare Effectiveness Data and Information Set (HEDIS), Epic Cosmos, the Population-Based Research to Optimize the Screening Process (PROSPR) consortium, the Health Center Program Uniform Data System (UDS), and the Breast Cancer Surveillance Consortium (BCSC). Additional secondary sources include commercial real-world data vendors, which typically require licensure and specialized analytic infrastructure (Figure 1).

Across sources, availability of screening data varies by cancer type, population, and calendar year (Figure 2). Many datasets report screening participation intermittently or for select malignancies only. Data from recent years (2020–2023) demonstrate substantial variation in reported screening rates across sources, reflecting differences in sampling frames, question wording, weighting approaches, and data processing methods (Figure 3).

### 3.2. Cancer Screening Rate Data Limitations and Resulting Impact

Across survey, EHR, and claims-based sources, four primary categories of data limitations were identified: data access, data accuracy, data consistency, and data completeness.

Data access limitations reflect fragmentation across data sources, governance structures, and access requirements. Survey datasets often require advanced statistical expertise to analyze, while visualization tools may limit analytic flexibility. EHR data are typically accessible only within individual health systems, and claims data frequently involve complex processing requirements, privacy protections, and licensing costs.

Data accuracy limitations were observed across all source types. Survey data are subject to recall bias, response bias, and representativeness challenges. EHR data may be affected by incomplete documentation, variability in clinical workflows, and human error. Claims data may include coding inaccuracies driven by billing practices rather than clinical intent. Key screening-related variables, such as smoking history, were inconsistently captured across datasets.

Data consistency limitations arise from changes in survey instruments, question wording, tumor types included, and data collection intervals. These variations complicate longitudinal analyses and cross-source comparisons (Figure 4 and Figure 5). In EHR and claims datasets, patient movement across providers or insurers contributes to fragmented longitudinal records (Figure 6).

Data completeness limitations include underrepresentation of certain populations, such as uninsured, rural, or underserved groups, and limited capture of downstream outcomes, including follow-up, cancer incidence, stage at diagnosis, and mortality. Across sources, gaps in demographic, socioeconomic, and risk-factor data restrict the ability to conduct stratified analyses.

### 3.3. Opportunities to Address Data Limitations

A multistakeholder workshop identified potential strategies to address limitations across data access, accuracy, consistency, and completeness. Proposed solutions varied by data source type and were qualitatively assessed based on feasibility and potential impact (Figure 7).

Proposed approaches to improve data access included development of centralized toolkits cataloging available data sources, publication of best-practice guidance, training initiatives to improve data literacy, expanded data-sharing agreements, and aggregation of EHR data into centralized or federated repositories.

Strategies to address data accuracy included simplifying and standardizing survey questions, piloting and validating survey instruments with advocacy organizations, transparent reporting of uncertainty measures, cross-validation across data sources, and quality assurance processes for claims-based data.

Proposals to enhance data completeness included establishing screening-focused registries, implementing EHR tools to capture key risk factors, advocating for additional screening-related billing codes, and supporting advanced data linkage efforts.

Approaches to improve data consistency included harmonizing survey instruments, mandating regular data collection, standardizing data elements, and improving longitudinal tracking capabilities within EHR systems.

## 4. Discussion

This study highlights persistent and interconnected limitations in the current landscape of cancer screening data, spanning survey-based, EHR, and claims-based sources. While these data are widely used to inform clinical guidance, policy decisions, advocacy efforts, and commercial research, challenges related to data access, accuracy, consistency, and completeness substantially constrain their interpretability and downstream utility [14,15].

A key finding supported by the secondary literature review and validated through expert interviews is that variation across data sources is often driven less by true differences in screening behavior and more by methodological differences in data collection, processing, and reporting. Differences in sampling frames, question wording, weighting strategies, and analytic assumptions can yield divergent screening rate estimates for the same cancer type, population, or time period. These discrepancies are particularly evident in recent years, during which disruptions in healthcare utilization and changes in screening recommendations further complicated interpretation. Without careful contextualization, such variability may be misinterpreted as meaningful changes in screening participation, potentially leading to misguided conclusions or policy responses [16].

Limitations in data access further exacerbate these challenges. Fragmentation across datasets, governance structures, and access requirements restricts the ability of researchers and policymakers to develop comprehensive, longitudinal views of screening participation. Survey datasets often require advanced statistical expertise, while visualization tools may trade analytic flexibility for usability. EHR and claims data, although rich in clinical detail, are typically siloed within health systems or proprietary infrastructures. EHR data are typically accessible only at the health system level, which limits their utility for population-wide analysis because it primarily reflects individuals who seek care within those systems. While initiatives such as the Rochester Epidemiology Project aggregate data across providers to approximate population-level coverage, this is likely not standard everywhere. To address this, increased data-sharing may improve access. Further, claims data presents further challenges due to complex processing requirements and strict protection protocols, resulting in high licensing costs. These barriers are especially difficult to overcome since much critical research relies on grant funding. As a result, analyses frequently reflect convenience rather than completeness, shaping conclusions based on what data are most accessible rather than what is most representative [17,18].

Data accuracy and completeness limitations have important implications for health equity. Underserved populations, including individuals who are uninsured, underinsured, or living in rural settings, are often underrepresented or inconsistently captured across screening data sources. Selection biases and incomplete data may mask or exacerbate disparities in screening access and outcomes. In addition, key risk factors and demographic variables are frequently missing or inconsistently documented, limiting the ability to stratify analyses and identify disparities. When screening estimates lack sufficient granularity, efforts to design targeted interventions or evaluate equity-focused policies are hindered. In order to drive support around new initiatives such as mobile screening units, community health center integration, culturally tailored survey instruments, or partnerships with community-based organizations, data for underserved populations must be complete and accurate. In this context, high-level screening rates may obscure meaningful variation across subpopulations rather than illuminate it.

The inability of most existing datasets to capture the full continuum of care, from risk assessment through screening, follow-up, diagnosis, and outcomes, further limits their utility. Because many data sources are not designed specifically for longitudinal screening evaluation, stakeholders often rely on proxy measures, extrapolation, or linkage across multiple datasets. While such approaches can provide valuable insights, they also introduce uncertainty and may amplify inconsistencies across sources. These limitations are particularly consequential when screening data are used to support guideline development, reimbursement decisions, or evaluation of emerging screening technologies.

Importantly, the findings underscore that no single data source can fully address current cancer screening data challenges in isolation. Instead, many limitations stem from fragmentation across the broader screening data ecosystem, including variability in data governance, collection methods, and reporting practices. Solutions identified through the multistakeholder workshop reflect the need for coordinated approaches that improve accessibility, standardization, timeliness, and transparency across data types. Near-term opportunities include harmonizing survey instruments, improving clinical documentation practices, and developing data-use toolkits to increase awareness of available resources. More resource-intensive strategies, such as the creation of screening-focused registries and improved longitudinal data integration, may offer greater long-term impact but require sustained collaboration, investment, and alignment across stakeholders.

Across limitation categories, several cross-cutting strategies emerged as particularly relevant and were prioritized based on an assessment of impact and feasibility. For data inaccessibility, publishing data toolkits and hosting educational workshops may improve usability and awareness among researchers and decision-makers [19]. Addressing data inaccuracy will require refining data collection and reporting practices, increasing transparency around uncertainty through the use of disclaimers and confidence intervals, and strengthening validation efforts [20]. For data inconsistency, standardizing reporting practices, mandating regular data collection, and ensuring timely dataset release may reduce year-to-year variation and misinterpretation [21]. Finally, improving data completeness will depend on expanding inclusion of underserved populations and developing tools to capture more detailed demographic, clinical, and risk-factor information across screening data sources [17].

Finally, this study has several important limitations. First, only a subset of the broad community was engaged during this project. The project focused heavily on US data and would benefit from additional global perspectives in subsequent work. While the 20-expert multistakeholder group allowed for a rich discussion, further engagement and work are needed to more accurately assess the impact and solutions that are most meaningful to the full landscape of cancer screening data stakeholders. Finally, it is fair to note that the overarching data limitation categories discussed in this study may be well-recognized across many areas of health services research. While the results of this study may not be groundbreaking, it is important to recognize that these limitations are not often widely appreciated beyond the research community that spends significant time working with this data. Ultimately, improving the quality and usability of cancer screening data is essential for ensuring that screening policies, screening surveillance systems, clinical guidance, and innovation efforts are grounded in accurate and equitable evidence.

We plan to follow this study with the creation of a future multistakeholder working group dedicated to the further investigation, broader recommendation development, prioritization, and execution of the highest-priority solutions identified here, and we encourage any interested parties to engage with the authors. It is our hope that implementation of some of these prioritized recommendations may improve future cancer screening surveillance, required policy development, and implementation of precision prevention initiatives. By prioritizing collaboration among data aggregators, clinicians, researchers, policymakers, and patient advocates, the cancer screening community can better align data infrastructure with the goal of improving early detection and reducing cancer-related morbidity and mortality.

## Figures and Tables

**Figure 1:**
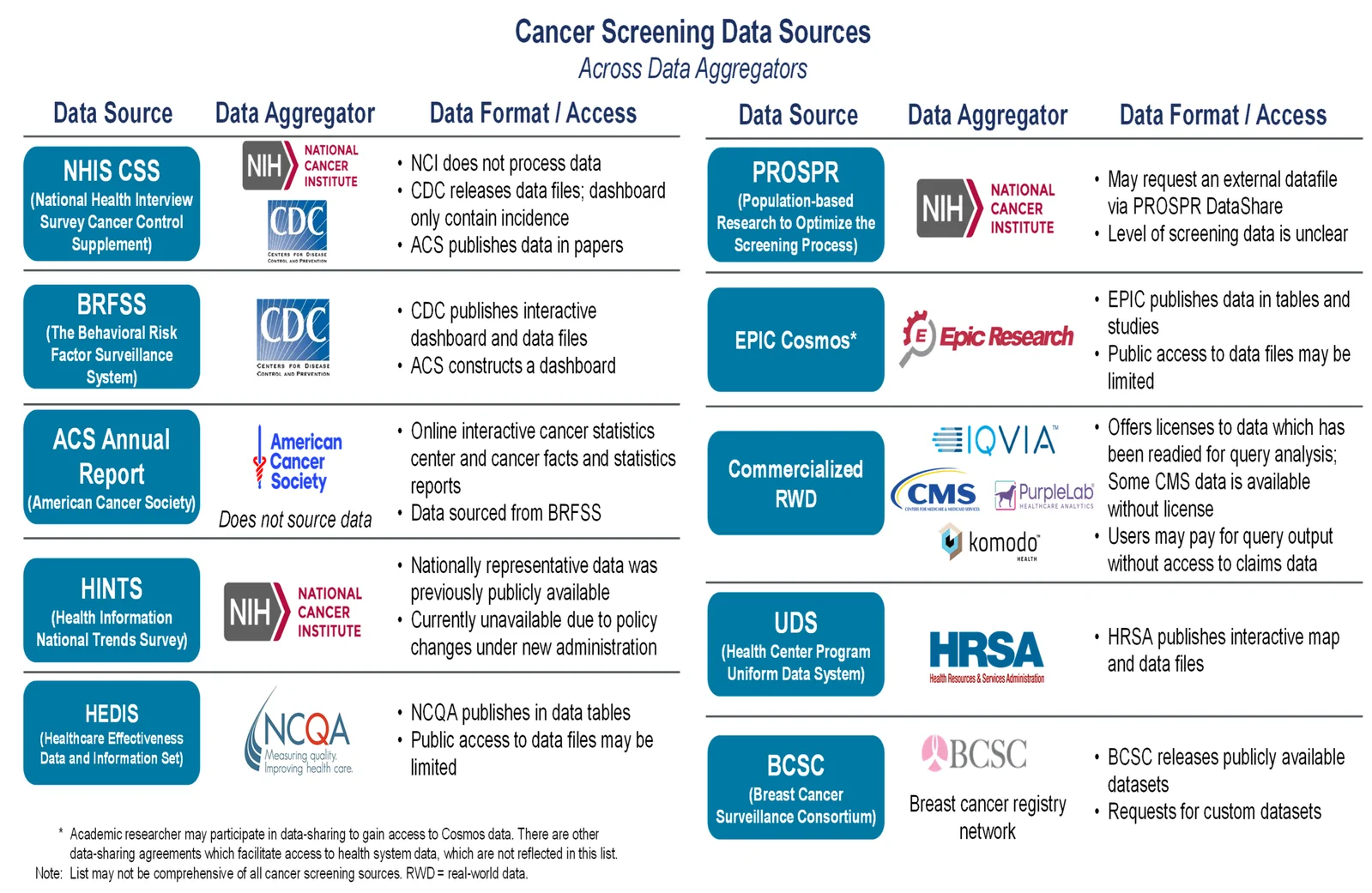
Cancer Screening Rate Data Sources: Multiple sources of primary and secondary clinical data inform cancer screening rates, including national surveys, electronic health records, claims, and various datasets from aggregators.

**Figure 2:**
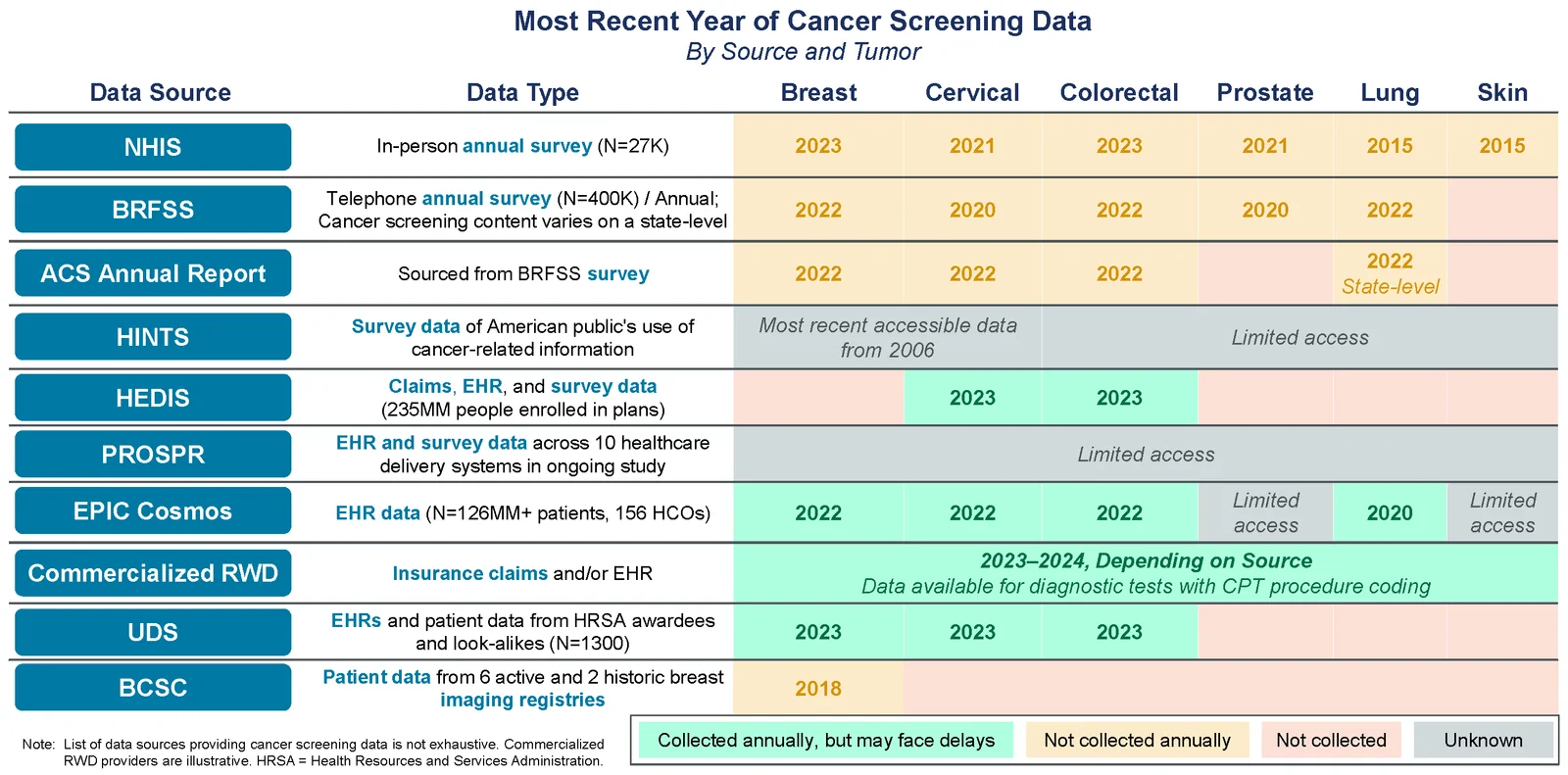
Tumor Type and Year Coverage Across Data Sources: Major gaps exist in annual coverage of tumor types across existing sources.

**Figure 3:**
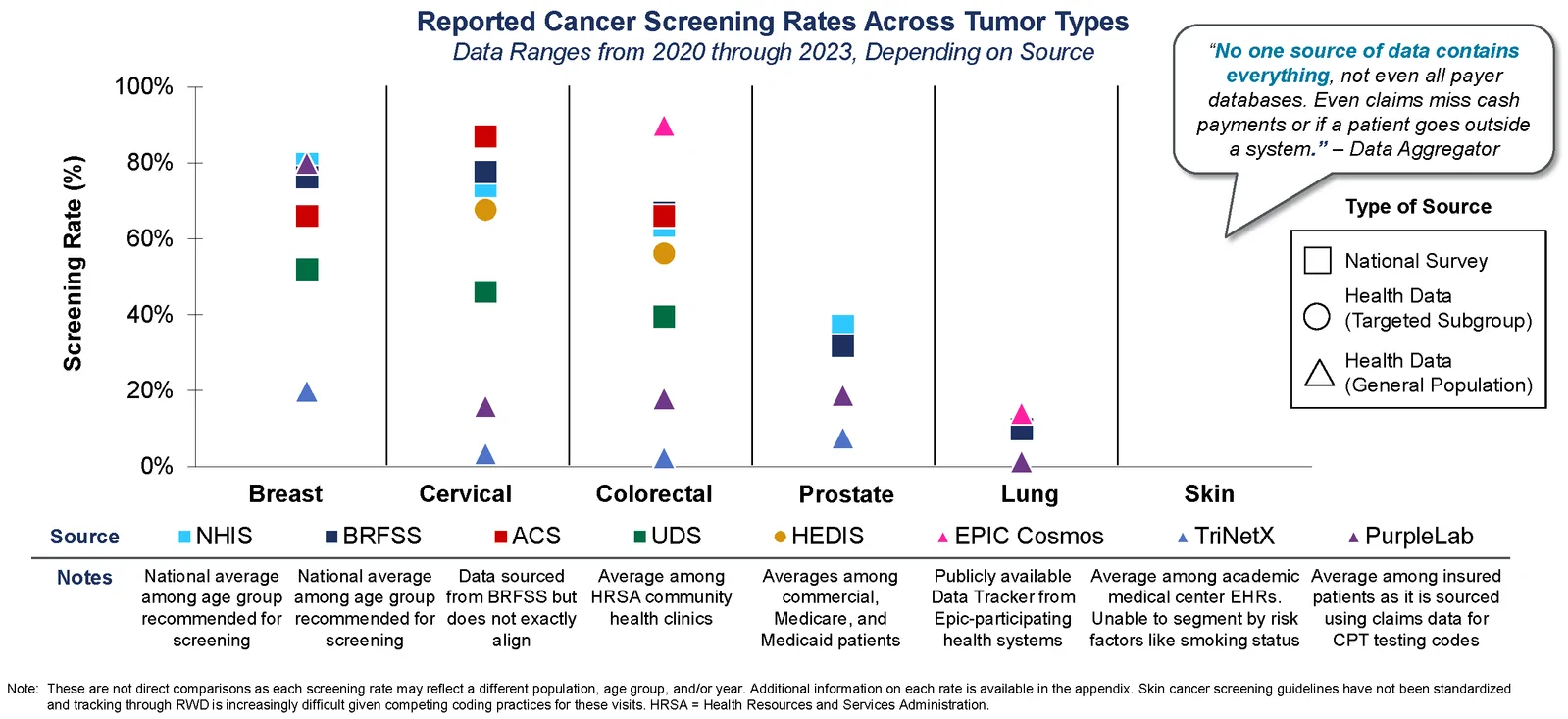
Cancer Screening Rate Inconsistencies Across Data Sources: Among more recent data, there are major discrepancies in reported screening rates. In part, this is driven by different sampling and processing methods.

**Figure 4:**
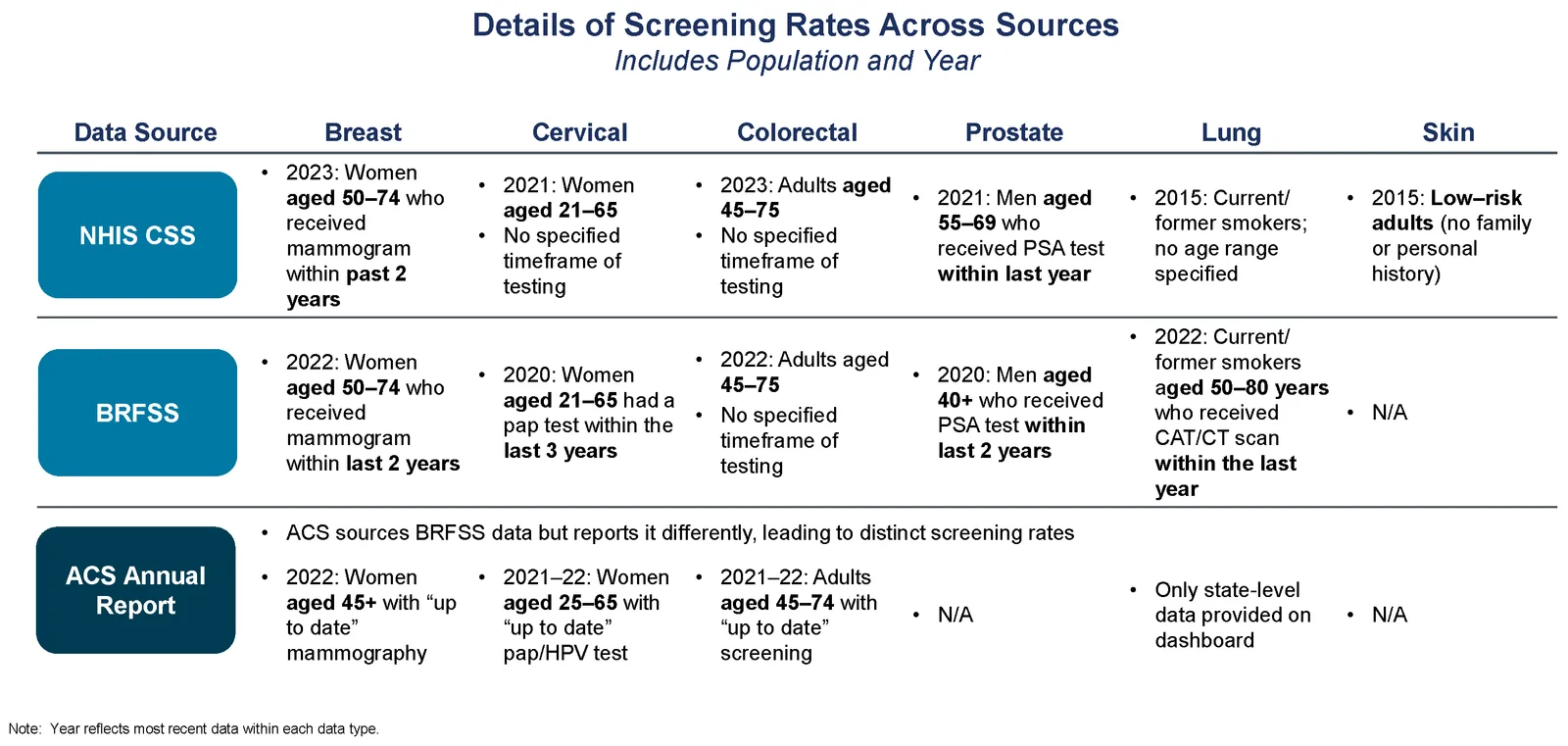
Survey-Based Cancer Screening Rate Data Collection Inconsistencies: Misaligned screening rate survey data is driven in part by different reporting years and age ranges.

**Figure 5:**
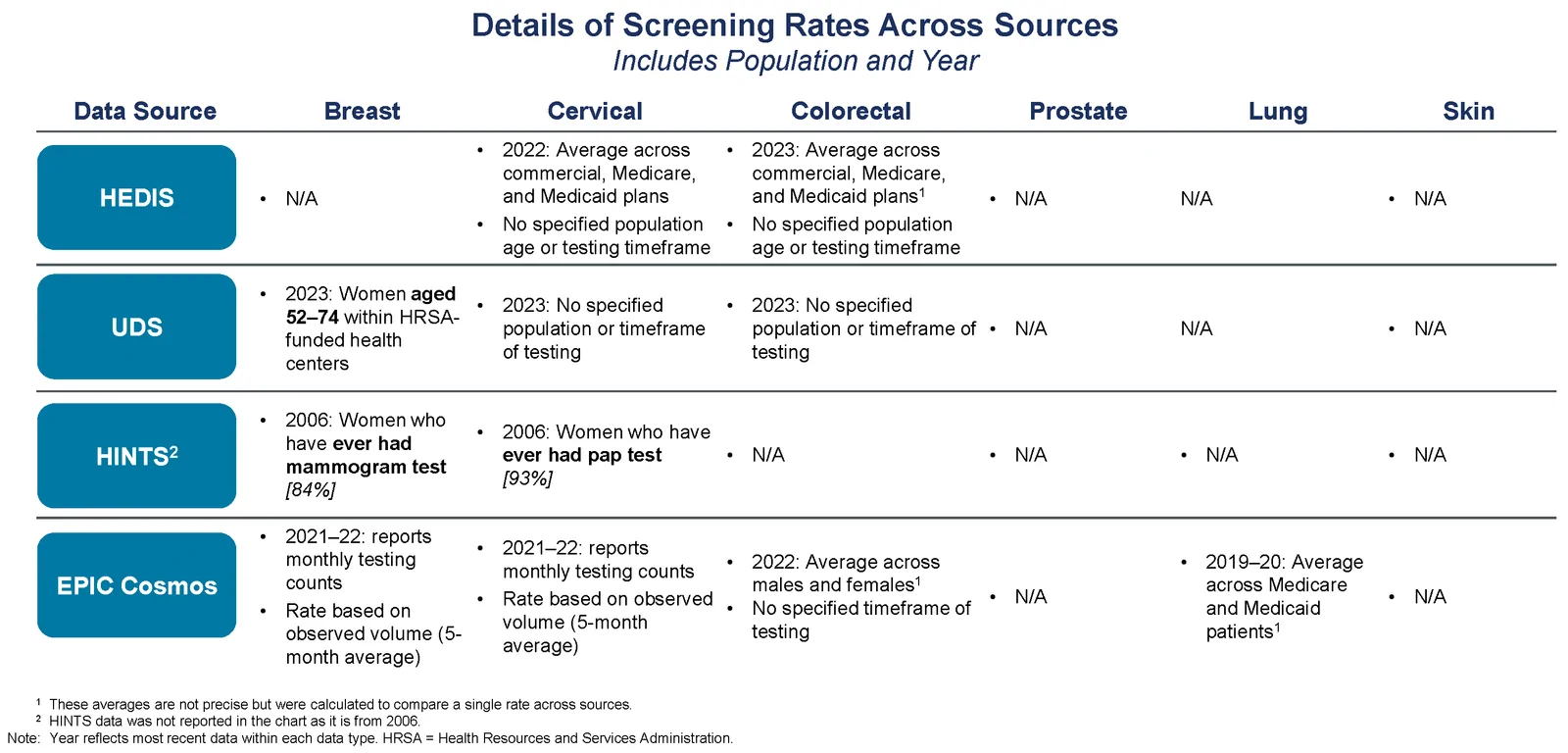
Claims/EHR-Based Cancer Screening Rate Data Collection Inconsistencies: Alternative data sources also have different data collection methods and reporting, which make comparisons challenging.

**Figure 6:**
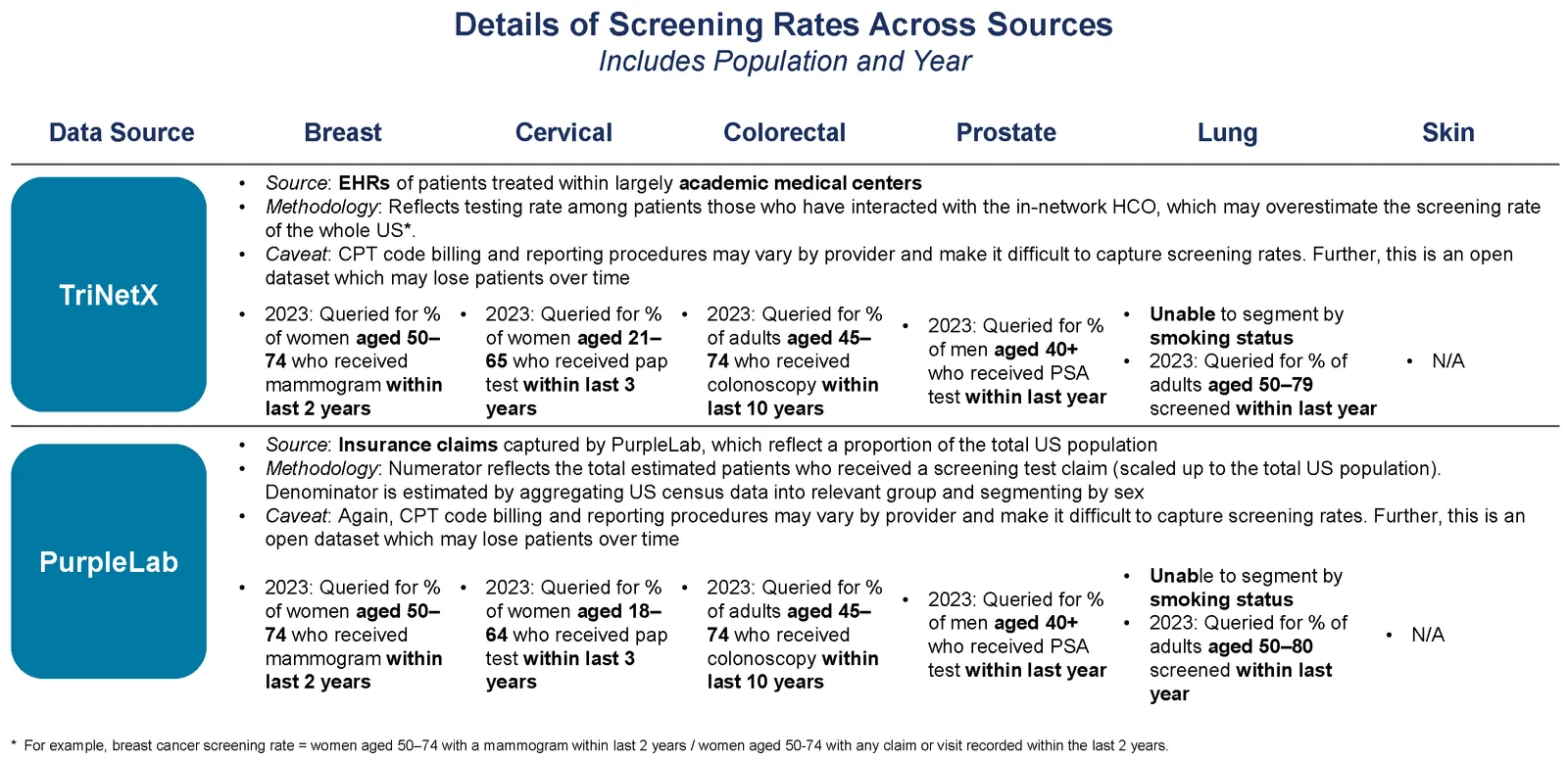
RWD-Based Cancer Screening Rate Data Collection Inconsistencies: Different methods exist to query RWD, which contribute to variable screening rate data.

**Figure 7:**
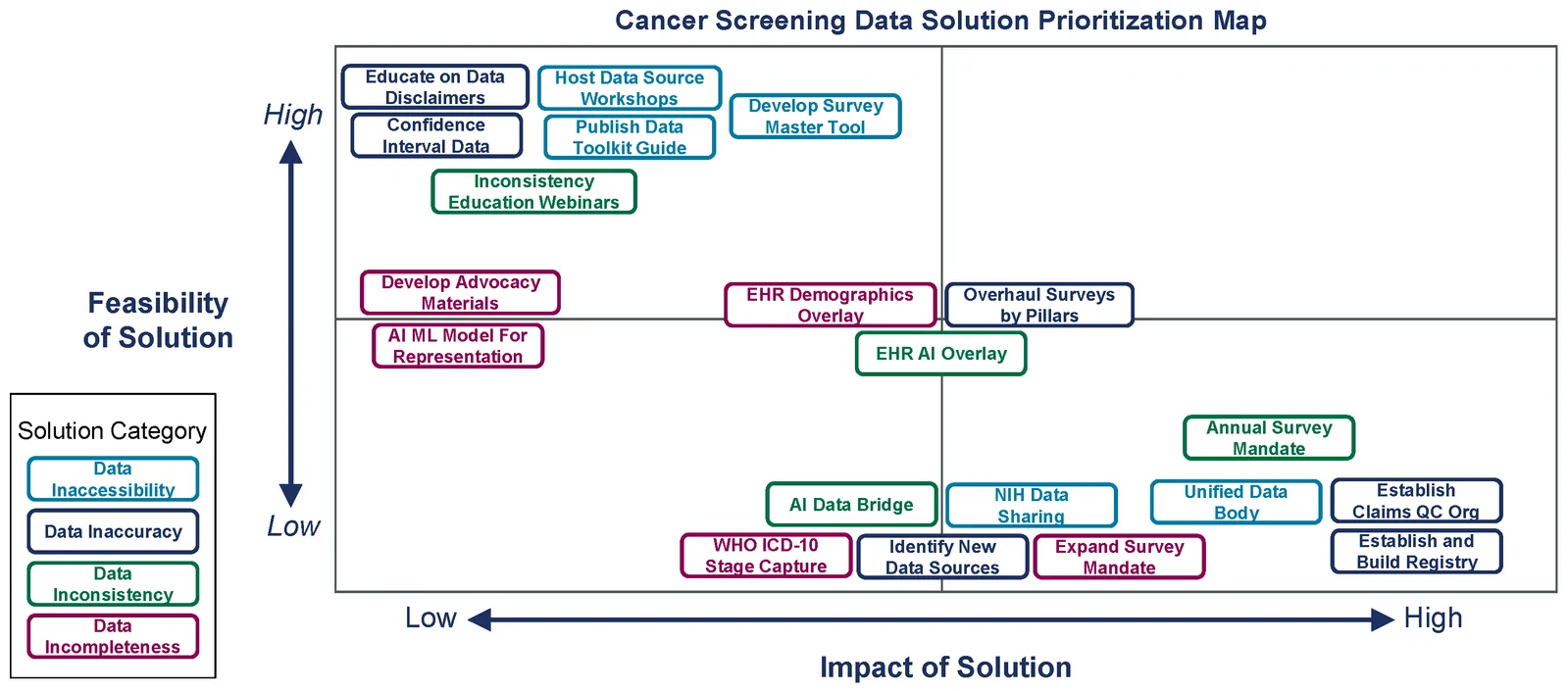
Cancer Screening Rate Data Solution Prioritization Map: Solutions to address the limitations of current cancer screening data were prioritized based on their impact and feasibility.

## Data Availability

The data underlying this article will be shared upon reasonable request to the corresponding author.

## References

[1] Aguiar-Ibáñez R., Mbous Y., Sharma S., Chakali R., Chawla E. (2025). Barriers to cancer screening uptake and approaches to overcome them: A systematic literature review. Front. Oncol..

[2] Pradhan P., Iyer H.S., Rebbeck T.R. (2025). Geographic and Temporal Patterns of Screening for Breast, Cervical, and Colorectal Cancer in the US, 1997–2019. JAMA Netw. Open.

[3] Maki K.G., Tan N.Q.P., Volk R.J., Toumazis I. (2025). Examining Lung Cancer Screening Uptake in the United States: Recent Research and Limitations of Public-Use Data. Cancer Epidemiol. Biomark. Prev..

[4] Rai A., Doria-Rose V.P., Silvestri G.A., Yabroff K.R. (2019). Evaluating Lung Cancer Screening Uptake, Outcomes, and Costs in the United States: Challenges With Existing Data and Recommendations for Improvement. J. Natl. Cancer Inst..

[5] ACS (2025). Cancer Surveillance Programs and Data Tracking.

[6] Penberthy L.T., Rivera D.R., Lund J.L., Bruno M.A., Meyer A.M. (2022). An overview of real-world data sources for oncology and considerations for research. CA Cancer J. Clin..

[7] Benavidez G.A., Sedani A.E., Felder T.M., Asare M., Rogers C.R. (2024). Rural-urban disparities and trends in cancer screening: An analysis of Behavioral Risk Factor Surveillance System data (2018–2022). JNCI Cancer Spectr..

[8] Gu T., Yuan J., White-Means S., Li M. (2024). Disparities and Gaps in Breast Cancer Screening for Women Aged 40 to 49 Years. JAMA Netw. Open.

[9] Althubaiti A. (2016). Information bias in health research: Definition, pitfalls, and adjustment methods. J. Multidiscip. Healthc..

[10] McGuckin T., Crick K., Myroniuk T.W., Setchell B., Yeung R.O., Campbell-Scherer D. (2022). Understanding challenges of using routinely collected health data to address clinical care gaps: A case study in Alberta, Canada. BMJ Open Qual..

[11] Campbell R., Ju A., King M.T., Rutherford C. (2022). Perceived benefits and limitations of using patient-reported outcome measures in clinical practice with individual patients: A systematic review of qualitative studies. Qual. Life Res..

[12] Division of Cancer Prevention and Control (2018). Measuring Breast, Cervical, and Colorectal Cancer Screening Rates in Health System Clinics. https://www.google.com/url?sa=t&rct=j&q=&esrc=s&source=web&cd=&ved=2ahUKEwiqzabah8CRAxWFFlkFHWdeFkcQFnoECBkQAQ&url=https%3A%2F%2Fstacks.cdc.gov%2Fview%2Fcdc%2F43398%2Fcdc_43398_DS1.pdf%3F&usg=AOvVaw3VTPpXinV8uoupVb6cHOzK&opi=89978449.

[13] American Cancer Society (2025). Quality Improvement Implementation Manual: Cancer Prevention and Screening. https://www.mysocietysource.org/Resource/2025%20ACS%20QI%20Implementation%20Manual.pdf.

[14] Ghalavand H., Shirshahi S., Rahimi A., Zarrinabadi Z., Amani F. (2024). Common data quality elements for health information systems: A systematic review. BMC Med. Inform. Decis. Mak..

[15] Lighterness A., Adcock M., Scanlon L.A., Price G. (2024). Data Quality-Driven Improvement in Health Care: Systematic Literature Review. J. Med. Internet Res..

[16] Goldman D., Lakdawalla D., Van Nuys K. (2023). Cancer-Related Technologies Have Changed a Lot. So Should Cancer Screening. USC Schaeffer Institute for Public Policy & Government Service. https://schaeffer.usc.edu/wp-content/uploads/2024/10/MCED_White_Paper.pdf.

[17] Trentham-Dietz A., Corley D.A., Del Vecchio N.J., Greenlee R.T., Haas J.S., Hubbard R.A., Hughes A.E., Kim J.J., Kobrin S., Li C.I. (2023). Data gaps and opportunities for modeling cancer health equity. J. Natl. Cancer Institute. Monogr..

[18] Joung R.H., Mullett T.W., Kurtzman S.H., Shafir S., Harris J.B., Yao K.A., Bilimoria K.Y., Cance W.G., Nelson H., Return-to-Screening Quality Improvement Collaborative (2022). Evaluation of a National Quality Improvement Collaborative for Improving Cancer Screening. JAMA Netw. Open.

[19] IEEE DataPort Strategies for Improving Data Accessibility in Academic Institutions. Strategies for Improving Data Accessibility in Academic Institutions | IEEE DataPort. https://ieee-dataport.org/news/strategies-improving-data-accessibility-academic-institutions.

[20] National Cancer Institute—Surveillance, Epidemiology, and End Results Program. Process—SEER Quality Improvement. SEER. https://seer.cancer.gov/qi/process.html.

[21] Aguinis H., Hill N.S., Bailey J.R. (2021). Best practices in data collection and preparation: Recommendations for reviewers, editors, and authors. Organ. Res. Methods.

